# Anti-staphylococcal activity of a polyphenol-rich citrus extract: synergy with β-lactams and low proficiency to induce resistance

**DOI:** 10.3389/fmicb.2024.1415400

**Published:** 2024-07-03

**Authors:** Diletta Mazzantini, Mariacristina Massimino, Marco Calvigioni, Virginia Rossi, Francesco Celandroni, Antonella Lupetti, Giovanna Batoni, Emilia Ghelardi

**Affiliations:** Department of Translational Research and New Technologies in Medicine and Surgery, University of Pisa, Pisa, Italy

**Keywords:** cytotoxicity, polyphenols, resistance development, staphylococci, synergy with β-lactams

## Abstract

**Introduction:**

Antibiotic resistance represents one of the most significant threats to public health in the 21st century. Polyphenols, natural molecules with antibacterial activity produced by plants, are being considered as alternative antimicrobial strategies to manage infections caused by drug-resistant bacteria. In this study, we investigated the antibacterial activity of a polyphenol mixture extracted from citrus fruits, against both antibiotic-susceptible and resistant strains of *Staphylococcus aureus* and *Staphylococcus epidermidis*.

**Methods:**

Broth microdilution and time-kill curve experiments were used to test the extract anti-staphylococcal activity. Cytotoxicity was assessed by the hemolysis assay. The interaction between the mixture and antibiotics was investigated by the checkerboard assay. The effect of B alone and in combination with oxacillin on the membrane potential was investigated by the 3,3′-dipropylthiadicarbocyanine iodide assay. The ability of the extract to induce the development of resistance was verified by propagating *S. aureus* for 10 transfers in the presence of sub-inhibitory concentrations.

**Results:**

The citrus extract was found to be active against all *Staphylococcus* strains at remarkably low concentrations (0.0031 and 0.0063%), displaying rapid bactericidal effects without being toxic on erythrocytes. In particular, B was found to rapidly cause membrane depolarization. When combined with methicillin, meropenem, and oxacillin, the mixture displayed synergistic activity exclusively against methicillin-resistant strains. We additionally show that the sequential exposure of *S. aureus* to sub-inhibitory concentrations did not induce the development of resistance against the extract.

**Discussion:**

Overall, these findings support the potential use of the citrus extract as promising option to manage staphylococcal infections and suggest that it may counteract the mechanism behind methicillin-resistance.

## 1 Introduction

More than 90 years from the discovery of penicillin by Alexander Fleming, the increasing use of antibiotics in the clinical practice, agriculture, veterinary, as well as livestock, progressively pushed the development and spreading of antibiotic resistance in bacteria (Ding et al., 2023). In fact, for ensuring their own survival, microbes can become resistant to drugs due to spontaneous mutations, but can also acquire resistance by receiving exogenous DNA through horizontal gene transfer (Munita and Arias, 2016; Zhu et al., 2023). Nowadays, antibiotic resistance represents a global health emergency that drastically restricts the spectrum of usable antibiotics and leads some infections to be untreatable with the forecast of 10 million deaths per year globally by 2050 (Tang et al., 2023).

Among Gram-positive bacteria, *Staphylococcus aureus* and *Staphylococcus epidermidis* are among the most frequently isolated microbes from hospital or community-acquired infections. These bacteria are known to be responsible for ocular, skin, wound, soft-tissue, urinary and respiratory-tract, implant-associated, and systemic infections, as well as endocarditis (Natsis and Cohen, 2018; Cheung et al., 2021; Lisowska-Łysiak et al., 2021; Severn and Horswill, 2023). The World Health Organization recently included *S. aureus* in the group of pathogens with the highest priority status due to its high degree of resistance to antibiotics (Mancuso et al., 2021). Many *S. aureus* strains are now characterized by methicillin-resistance (MRSA) that confers resistance to almost all β-lactam antibiotics (Peacock and Paterson, 2015). MRSA strains frequently show resistance to other classes of antibiotics, being often multidrug-resistant (MDR) or even pandrug-resistant (Mancuso et al., 2021). *Staphylococcus epidermidis* strains resistant to methicillin (MRSE) and other drug classes are also frequently isolated (Severn and Horswill, 2023).

Due to the high rate of bacterial resistance to existing antibiotics and to the difficulty in discovering additional molecular targets for manufacturing new drugs, the scientific interest is progressively focusing on the development of novel antimicrobial strategies to face up antibiotic-resistant infections (Chang et al., 2022; Cook and Wright, 2022; Ruggieri et al., 2023). In this view, plant-derived molecules with antimicrobial activity have been highlighted as promising and appealing alternatives or complements to antibiotics (Murugaiyan et al., 2022). Among phytochemicals, polyphenols are abundant in fruits and are constituted by an aromatic ring with one or more hydroxyl groups. Based on their structure, polyphenols are grouped in several classes (i.e., phenolic acids, flavonoids, lignans, stilbenes) and subclasses (Lobiuc et al., 2023). These compounds are known to provide many beneficial effects on human health, including antioxidant, anti-inflammatory, anti-diabetic, anti-cancer, as well as to possess cardio-, osteo-, and neuroprotective activities (Rana et al., 2022; Roszkowski, 2023; William Raja et al., 2023). A great number of reports highlighting the antimicrobial activity of polyphenols, particularly flavonoids, populates the literature, thus significantly increasing the interest toward these molecules (Álvarez-Martínez et al., 2020; Manso et al., 2021; Montenegro-Landívar et al., 2021; Khomsi et al., 2022; Konputtar et al., 2022; Oulahal and Degraeve, 2022). Although the antimicrobial activity of polyphenols could be extremely variegated, most molecules exert their antimicrobial effects by disrupting the bacterial plasma membrane, thus leading to the loss of cellular content and cell membrane depolarization (Bouarab Chibane et al., 2019; Nassarawa et al., 2022; Liu et al., 2023). Some polyphenols have been additionally shown to penetrate in the cytoplasm, interfere with bacterial metabolism, and affect DNA, RNA, and protein synthesis and functions (Oulahal and Degraeve, 2022; Lobiuc et al., 2023). Due to their properties, these molecules have been recently proposed as natural food preservatives and biocides in the food industry (Oulahal and Degraeve, 2022; Ullah et al., 2022). Polyphenols extracted from different vegetables displayed marked antimicrobial activity against *S. aureus* and *S. epidermidis*, being for this reason proposed as new anti-staphylococcal therapeutic options (Álvarez-Martínez et al., 2020; Abedini et al., 2021; Pirzadeh et al., 2021; Guimarães et al., 2023).

This *in vitro* study aimed at investigating the activity of a polyphenol mixture extracted from citrus fruits against *S. aureus* and *S. epidermidis*, which frequently display antibiotic-resistant profile and are responsible for a variety of human infections. Particular emphasis was given to the potential interaction of the extract with commercially available antibiotics and to its cytotoxicity on human erythrocytes. Considering the ability of *Staphylococcus* spp. to rapidly develop drug resistance, we additionally evaluated the evolution of resistance to the mixture in *S. aureus*, which was selected as model organism for clinically relevant staphylococci.

## 2 Materials and methods

### 2.1 Bacterial strains, chemicals, and culture conditions

In this study, *S. aureus* ATCC 6538, *S. aureus* ATCC 43300, *S. epidermidis* ATCC 35984, and two *S. epidermidis* clinical isolates (named CI-1 and CI-2) were used (Mazzantini et al., 2024). All strains were propagated on Mueller-Hinton Agar (MHA; Thermo Fisher Scientific, Waltham, United States) at 37°C for 24 h. For the antimicrobial susceptibility assays by broth microdilution, cation-adjusted Mueller-Hinton Broth (CAMHB; Merck KGaA, Darmstadt, Germany) containing 20–25 mg/L of calcium and 10–12.5 mg/L of magnesium was used (European Committee on Antimicrobial Susceptibility Testing, 2022). Biosecur® F440D-K, from now on referred to as B, was kindly provided by OFF-HEALTH S.p.A. (Florence, Italy). B is a homogeneous mixture mainly constituted by polyphenols and extracted from the edible parts, including the albedo components, of *Citrus aurantium amara*, *Citrus reticulata*, and *Citrus sinensis* (U.S. Food and Drug Administration, 2013). In particular, B contains 2.70–5.00% of total polyphenols, of which 0.50–1.20% flavonoids, such as at least rutin, hesperidin, and quercetin, and 57–64% glycerin (U.S. Food and Drug Administration, 2013). Methicillin sodium salt, meropenem trihydrate, levofloxacin, and chloramphenicol powders were purchased from Thermo Fisher Scientific (Waltham, USA). Oxacillin sodium salt and gentamycin were purchased from Merck KGaA (Darmstadt, Germany). Drugs were dissolved in suitable solvents as indicated by the manufacturers. 3,3′-dipropylthiadicarbocyanine iodide (DiSC_3_(5)) was acquired from Merck KGaA (Darmstadt, Germany) and dissolved in dimethyl sulfoxide (DMSO; Carlo Erba, Milan, Italy) at a final concentration of 100 μM.

### 2.2 Susceptibility testing

Bacterial strains were subjected to antibiotic susceptibility testing by broth microdilution (European Committee on Antimicrobial Susceptibility Testing, 2024a). Briefly, bacterial suspensions were prepared by suspending a freshly grown colony on MHA in sterile saline solution (0.85% NaCl) to a density of 0.5 McFarland (corresponding to ~1–2 × 10^8^ CFU/mL). Bacteria were diluted in CAMHB to a final concentration of ~5 × 10^5^ CFU/mL and 100 μL were inoculated in each well of ITGP100 panels (Bruker Daltonics GmbH, Bremen, Germany). The antibiotics tested in the panels were ampicillin, ampicillin/sulbactam, cefoxitin, ceftarolin, ceftobiprole, clindamycin, dalbavancin, daptomycin, doxycycline, erythromycin, erythromycin/clindamycin, fusidic acid, gentamycin, gentamycin high level 128, levofloxacin, linezolid, moxifloxacin, mupirocin, nitrofurantoin, oxacillin, rifampicin, streptomycin high level 512, tedizolid, teicoplanin, tigecycline, trimethoprim/sulfamethoxazole, and vancomycin. Panels were incubated at 35 ± 1°C for 18 ± 2 h according to manufacturer instructions. Minimal inhibitory concentration (MIC) was defined as the lowest concentration of antimicrobial agent that completely inhibited visible growth and determined following the EUCAST reading guide for broth microdilution (version 5.0; European Committee on Antimicrobial Susceptibility Testing, 2024b). Susceptibility categories (S, susceptible, standard dosing regimen; I, susceptible, increased exposure; R, resistant) were defined based on the Breakpoint Tables for *Staphylococcus* spp. (version 14.0; European Committee on Antimicrobial Susceptibility Testing, 2024a). Experiments were repeated three times in separate days.

### 2.3 Evaluation of the minimal inhibitory concentration and minimal bactericidal concentration

Before each experiment, B was freshly diluted to a final concentration of 0.2% (v/v) in CAMHB and immediately used for the assays. B was two-fold serially diluted in CAMHB in 96-well microplates (Carlo Erba, Milan, Italy) to obtain a final volume of 100 μL per well. The concentrations of the polyphenol mixture used in the assays ranged from 0.10 to 0.000049%. Bacterial suspensions were prepared as described above, diluted in CAMHB to a final concentration of ~5 × 10^5^ CFU/mL, and 100 μL inoculated in wells of 96-well microplates. In parallel, wells containing bacteria in CAMHB and sterile CAMHB were used as positive and negative controls, respectively. Microplates were incubated at 35 ± 1°C for 18 ± 2 h and the MIC of B determined (European Committee on Antimicrobial Susceptibility Testing, 2022, 2024a). Determination of the Minimal Bactericidal Concentration (MBC) was performed by plating 100 μL of the suspensions taken from wells with the MIC and with concentrations of the extract higher than the MIC. MBC was defined as the lowest concentration of B killing at least 99.9% of viable microbes. Plates were incubated at 37°C for 24 h. Three independent biological replicates with two technical replicates each were performed.

### 2.4 Time-kill curves

Time-kill assay was carried out using *S. aureus* ATCC 6538 and *S. epidermidis* CI-1 as model organisms, as previously described (Mencucci et al., 2022). Briefly, microbes were suspended in 0.01 M Sodium Phosphate Buffer (NaPB, pH 7.2) to a density of 0.5 McF and about 10^6^ CFU inoculated in NaPB containing the extract at the MIC value (0.0031% for *S. aureus* ATCC 6538 and 0.0063% for *S. epidermidis* CI-1). As control, bacteria were inoculated in NaPB alone and NaPB containing oxacillin at the MIC values (i.e., 0.063 and 0.125 mg/L for *S. aureus* ATCC 6538 and *S. epidermidis* CI-1, respectively). Suspensions were incubated at 37°C for up to 1 h and 100 μL subjected to plate count after 1, 10, 45, and 60 min of incubation. Plates were incubated at 37°C for 24 h. The percentage of survival was calculated using the following formula: (number of CFUs recovered/number of CFUs inoculated) × 100. Experiments were repeated three times in separate days. Quantitative data were expressed as the mean ± standard deviation (SD).

### 2.5 Hemolysis assay

Hemolysis assay was performed following the protocol recently proposed by Sæbø et al. (2023). Human red blood cells (RBCs) were obtained from blood samples collected at the Clinical Microbiology Laboratory of the Pisa University Hospital for serological examinations. Anonymous residual samples, typically discarded after clinical procedures, were used to collect RBCs that were diluted to 1% in Phosphate Buffered Saline (PBS, pH 7.0). For this type of study, no informed consent was required. Aliquots (50 μL) of the 1% RBCs solution were transferred into 96-well round bottom microplates (Carlo Erba, Milan, Italy) and mixed with 50 μL of B solutions at concentrations ranging from 0.05 to 0.00039% (v/v). RBCs mixed with PBS and RBCs mixed with 10% Triton X-100 (Merck KGaA, Darmstadt, Germany) were used as negative (0% lysis) and positive (100% lysis) controls in the assay, respectively. Microplates were incubated at 37°C for 60 min and centrifuged at 1,700 ×*g* for 5 min at 4°C. Aliquots of supernatants (50 μL) were transferred to a new 96-well flat bottom microplate (Carlo Erba, Milan, Italy) and the optical density at 405 nm (OD_405_) was measured in a Multiskan™ FC Microplate Photometer (Thermo Fisher Scientific, Waltham, United States). The percentage of hemolysis was calculated according to the formula: [(OD_405_ of the B suspension − OD_405_ of the negative control)/(OD_405_ of the positive control − OD_405_ of the negative control)] × 100. Three independent biological replicates with two technical replicates each were performed. Quantitative data were expressed as the mean ± SD.

### 2.6 Giemsa staining and microscopical observation of RBCs

Five microliter of RBCs taken from wells of the microplates used in the hemolysis assays were smeared on glass slides, air-dried, and fixed for 5 s with methanol (Merck KGaA, Darmstadt, Germany). Smears were stained by using the Giemsa Stain (modified) (TCS Biosciences Ltd., Buckingham, UK) according to manufacturer’s instructions and air-dried. Glass slides were observed at 1,000 × magnification using an optical microscope (BH-2; Olympus, Tokyo, Japan).

### 2.7 Checkerboard assay

The interaction between B and antibiotics belonging to different classes was investigated by the checkerboard assay in 96-well microplates (Carlo Erba, Milan, Italy) following the protocol described by Bellio et al. (2021). In particular, four β-lactams (i.e., ampicillin, methicillin, oxacillin, and meropenem), one glycopeptide (vancomycin), one fluoroquinolone (levofloxacin), one aminoglycoside (gentamycin) antibiotics, and two miscellaneous agents (chloramphenicol and daptomycin) (chloramphenicol were tested in combination with B). Each plate included wells containing CAMHB alone and CAMHB with bacteria as negative and positive controls, respectively. Microplates were incubated at 35 ± 1°C for 18 ± 2 h and MIC values determined. The fractional inhibitory concentration index (FICI) was calculated using the following formula: (MIC of B in combination)/(MIC of B alone) + (MIC of antibiotic in combination)/(MIC of antibiotic alone). The FICI values were interpreted as follows: FICI ≤0.5 indicate synergy, 0.5 < FICI <4 indicate indifference, while values >4 indicate antagonism (She et al., 2022). Three independent biological replicates were performed.

### 2.8 Assessment of membrane potential changes

The ability of B alone and in combination with oxacillin to cause changes in membrane potential was checked by the DiSC_3_(5) assay. *S. aureus* ATCC 43300 was grown in CAMHB at 37°C to OD_600_ of 0.25. Cells were collected, washed with HEPES buffer (5 mM HEPES, 20 mM glucose, pH 7.2), and suspended in HEPES buffer containing 100 mM KCl. The suspension was dispensed in 96-well black microplates and plates directly read using the Varioskan® LUX microplate reader (Thermo Fisher Scientific; excitation: 622 nm, emission: 670 nm). Then, 1 μM DiSC_3_(5) (Merck KGaA) was added and fluorescence measured. The suspension was incubated 1 h at 37°C in the dark for enabling DiSC_3_(5) uptake and fluorescence was recorded again for verifying dye quenching. B at the MIC and 2-fold the MIC (0.0063 and 0.0126%, respectively), oxacillin at the MIC (4 mg/L), and B in combination with oxacillin both at the MIC values were added. Untreated cells were used as negative controls of the assay. Fluorescence intensity was recorded after about 20 s from antimicrobial supplementation and was measured every 20 s for a total of 10 min.

### 2.9 Evolution of resistance to the citrus extract

The development of resistance to the extract was investigated in *S. aureus* ATCC 6538, following a protocol previously developed for dermatophytes (Mazzantini et al., 2021). Before performing experiments, the MIC and sub-inhibitory concentrations of B in MHA were determined by the agar dilution assay. Briefly, *S. aureus* (10^5^ CFU/plate) was seeded on MHA plates containing two-fold serial dilutions of the extract ranging from 0.05 to 0.0016% and on MHA plates without B as control. Plates were incubated at 37°C for 24 h. The MIC of the citrus mixture in MHA was defined as the lowest concentration preventing growth of macroscopically visible colonies on B-containing plates, when visible growth was present on the control plates. *S. aureus* (10^5^ CFU/plate) was serially propagated for 10 sequential transfers on MHA plates containing sub-inhibitory concentration of the extract. After the 5th and the 10th transfer, 10^10^ bacteria were collected and seeded on MHA plates containing 2-fold the MIC of B determined in agar dilution assay. In parallel, bacteria collected after the 10th transfer were subjected to broth microdilution assays to evaluate the emergence of resistance to β-lactams (ampicillin, methicillin, oxacillin, and meropenem). Experiments were repeated three times in separate days.

## 3 Results

### 3.1 Antibiotic resistance profile of *Staphylococcus* strains

All *Staphylococcus* strains were subjected to antibiotic susceptibility testing. As shown in Table 1, *S. aureus* ATCC 6538 and *S. epidermidis* CI-1 resulted susceptible to all the tested antibiotics. In contrast, *S. aureus* ATCC 43300 and *S. epidermidis* ATCC 35984 resulted resistant to clindamycin, erythromycin, gentamycin, and oxacillin. The susceptibility to levofloxacin at dosing regimen (S) or at increased exposure (I) of the four strains could not be established by using these panels due to the absence of growth at the lowest levofloxacin concentration (i.e., 1 mg/L). However, when broth microdilution was repeated with lower drug concentrations in the checkerboard assay (Table 2), these strains resulted to be I to levofloxacin based on the Breakpoint Tables for *Staphylococcus* spp. (version 14.0; European Committee on Antimicrobial Susceptibility Testing, 2024a). *S. epidermidis* CI-2 showed the highest profile of antibiotic resistance, since resistant to cefoxitin, clindamycin, erythromycin, fusidic acid, gentamycin, levofloxacin, moxifloxacin, oxacillin, teicoplanin, trimethoprim/sulfamethoxazole, and vancomycin.

**Table 1:** Antibiotic-resistance profiles of *Staphylococcus* strains included in the study.

	*S. aureus* ATCC 6538		*S. aureus* ATCC 43300		*S. epidermidis* ATCC 35984		*S. epidermidis* CI-1		*S. epidermidis* CI-2	
Antibiotic	MIC (mg/L)	Interpretation^a^	MIC (mg/L)	Interpretation^a^	MIC (mg/L)	Interpretation^a^	MIC (mg/L)	Interpretation^a^	MIC (mg/L)	Interpretation^a^
Ampicillin	≤0.25	NA	8	NA	>8	NA	≤0.25	NA	8	NA
Ampicillin/sulbactam	≤2	NA	8	NA	>8	NA	≤2	NA	≤2	NA
Cefoxitin	≤4	S	>8	R	>8	NA	≤4	NA	>8	NA
Ceftaroline	≤0.5	S	0.5	S	0.5	NA	≤0.25	NA	0.5	NA
Ceftobiprole	≤0.25	S	1	S	2	NA	≤0.25	NA	1	NA
Clindamycin	≤0.12	S	>1	R	>1	R	0.25	S	>1	R
Dalbavancin	≤0.06	S	≤0.06	S	≤0.06	S	≤0.06	S	≤0.06	S
Daptomycin	1	S	1	S	1	S	0.5	S	1	S
Doxycycline	≤0.5	S	<0.5	S	≤0.5	S	1	S	<0.5	S
Erythromycin	≤1	S	>4	R	>4	R	≤1	S	>4	R
Erythromycin/clindamycin	≤4/0.5	NA	>4/0.5	NA	>8/1.5	NA	4/0.5	NA	>4/0.5	NA
Fusidic Acid	0.5	S	<0.25	S	0.5	S	1	S	>1	R
Gentamycin	≤0.25	S	>4	R	>4	R	0.5	S	>4	R
Gentamycin high level 128	≤128	NA	≤128	NA	≤128	NA	≤128	NA	>128	NA
Levofloxacin	≤1	I or S	≤1	I or S	≤1	I or S	≤1	I or S	4	R
Linezolid	2	S	2	S	≤1	S	2	S	≤1	S
Moxifloxacin	≤0.125	S	≤0.12	S	≤0.125	S	≤0.125	S	>0.5	R
Mupirocin	≤1	NA	≤1	NA	≤1	NA	≤1	NA	≤1	NA
Nitrofurantoin	≤16	NA	≤16	NA	≤16	NA	≤16	NA	≤16	NA
Oxacillin	≤0.25	S	>2	R	>2	R	≤0.25	S	>2	R
Rifampicin	≤0.06	S	≤0.06	S	≤0.06	S	≤0.06	S	≤0.06	S
Streptomycin high level 512	≤512	NA	≤512	NA	>512	NA	≤512	NA	≤512	NA
Tedizolid	≤0.5	S	≤0.5	S	≤0.5	S	≤0.5	S	≤0.5	S
Teicoplanin	1	S	0.5	S	2	S	4	S	>4	R
Tigecycline	≤0.12	S	≤0.12	S	≤0.12	S	0.25	S	≤0.12	S
Trimetophrim/sulfametoxazole	≤0.25/4.75	S	≤0.25/4.75	S	≤2/38	S	≤0.25/4.75	S	>4/76	R
Vancomycin	2	S	0.5	S	2	S	2	S	>4	R

^a^MIC values were interpreted basing on Clinical Breakpoint Tables for *Staphylococcus* spp. (version 14.0; European Committee on Antimicrobial Susceptibility Testing, 2024a). S: susceptible, standard dosing regimen; I: susceptible, increased exposure; R: resistant; NA: breakpoints are not available.

**Table 2:** Interaction of B with antibiotics against *Staphylococcus* strains.

	B (%) alone	Antibiotic (mg/L) alone	B (%) in combination with antibiotic	Antibiotic (mg/L) in combination with B	MIC fold change B-antibiotic	FICI^a^
*S. aureus* ATCC 6538	0.0031	Ampicillin (0.031)	0.0031	0.031	–	2
		Methicillin (0.50)	0.0031	0.5	–	2
		Meropenem (0.016)	0.0031	0.016	–	2
		Oxacillin (0.063)	0.0031	0.063	–	2
		Levofloxacin (0.25)	0.0031	0.25	–	2
		Chloramphenicol (0.25)	0.0031	0.25	–	2
		Gentamycin (0.063)	0.0031	0.063	–	2
		Daptomycin (1)	0.0031	1	–	2
		Vancomycin (2)	0.0031	2	–	2
*S. aureus* ATCC 43300	0.0063	Ampicillin (8)	0.00078	2	8–4	**0.375**
		Methicillin (8)	0.00039	2	16–4	**0.312**
		Meropenem (2)	0.00078	0.5	8–4	**0.375**
		Oxacillin (4)	0.0016	0.25	4–16	**0.312**
		Levofloxacin (0.25)	0.0063	0.25	–	2
		Chloramphenicol (8)	0.0063	8	–	2
		Gentamycin (128)	0.0031	128	–	2
		Daptomycin (1)	0.0063	1	–	2
		Vancomycin (0.5)	0.0063	0.5	–	2
*S. epidermidis* ATCC 35984	0.0031	Ampicillin (32)	0.00078	8	4–4	0.5
		Methicillin (64)	0.00078	8	4–8	**0.375**
		Meropenem (16)	0.00078	2	4–8	**0.375**
		Oxacillin (32)	0.00078	4	4–8	**0.375**
		Levofloxacin (0.25)	0.0031	0.25	–	2
		Chloramphenicol (4)	0.0031	4	–	2
		Gentamycin (32)	0.0031	32	–	2
		Daptomycin (2)	0.0031	2	–	2
		Vancomycin (0.5)	0.0031	0.5	–	2
*S. epidermidis* CI-1	0.0063	Ampicillin (0.25)	0.0063	0.25	–	2
		Methicillin (2)	0.0063	2	–	2
		Meropenem (0.063)	0.0063	0.063	–	2
		Oxacillin (0.125)	0.0063	0.125	–	2
		Levofloxacin (0.25)	0.0063	0.25	–	2
		Chloramphenicol (4)	0.0063	4	–	2
		Gentamycin (1)	0.0063	1	–	2
		Daptomycin (0.5)	0.0063	0.5	–	2
		Vancomycin (2)	0.0063	2	–	2
*S. epidermidis* CI-2	0.0063	Ampicillin (8)	0.00078	2	8–4	**0.375**
		Methicillin (64)	0.00078	16	8–4	**0.375**
		Meropenem (8)	0.0016	1	4–8	**0.375**
		Oxacillin (64)	0.0016	8	4–8	**0.375**
		Levofloxacin (8)	0.0063	8	–	2
		Chloramphenicol (8)	0.0063	8	–	2
		Gentamycin (256)	0.0063	256	–	2
		Daptomycin (1)	0.0063	1	–	2
		Vancomycin (16)	0.0063	16	–	2

^a^Fractional Inhibitory Concentration Index. FICI values marked in bold indicate synergy.

Due to the resistance to oxacillin, *S. aureus* ATCC 43300 was defined as MRSA, while *S. epidermidis* ATCC 35984 and *S. epidermidis* CI-2 as MRSE. In addition, being resistant to at least one drug belonging to at least three different antibiotic classes (Magiorakos et al., 2012), *S. aureus* ATCC 43300, *S. epidermidis* ATCC 35984, and *S. epidermidis* CI-2 were also defined as MDR.

### 3.2 The citrus extract was active at very low concentrations against staphylococci

The anti-staphylococcal activity of B, a mixture of polyphenols extracted from three citrus species, was investigated. B showed marked antibacterial effect against both antibiotic-susceptible and -resistant staphylococci, with MIC values ranging from 0.0031% (for *S. aureus* ATCC 6538 and *S. epidermidis* ATCC 35984) to 0.0063% (for *S. aureus* ATCC 43300, *S. epidermidis* CI-1 and CI-2). No colonies were obtained by seeding aliquots from wells containing B at the MIC values (i.e., MIC = MBC), thus indicating that the extract exerts bactericidal effect against all the tested microbes.

**Figure 1:**
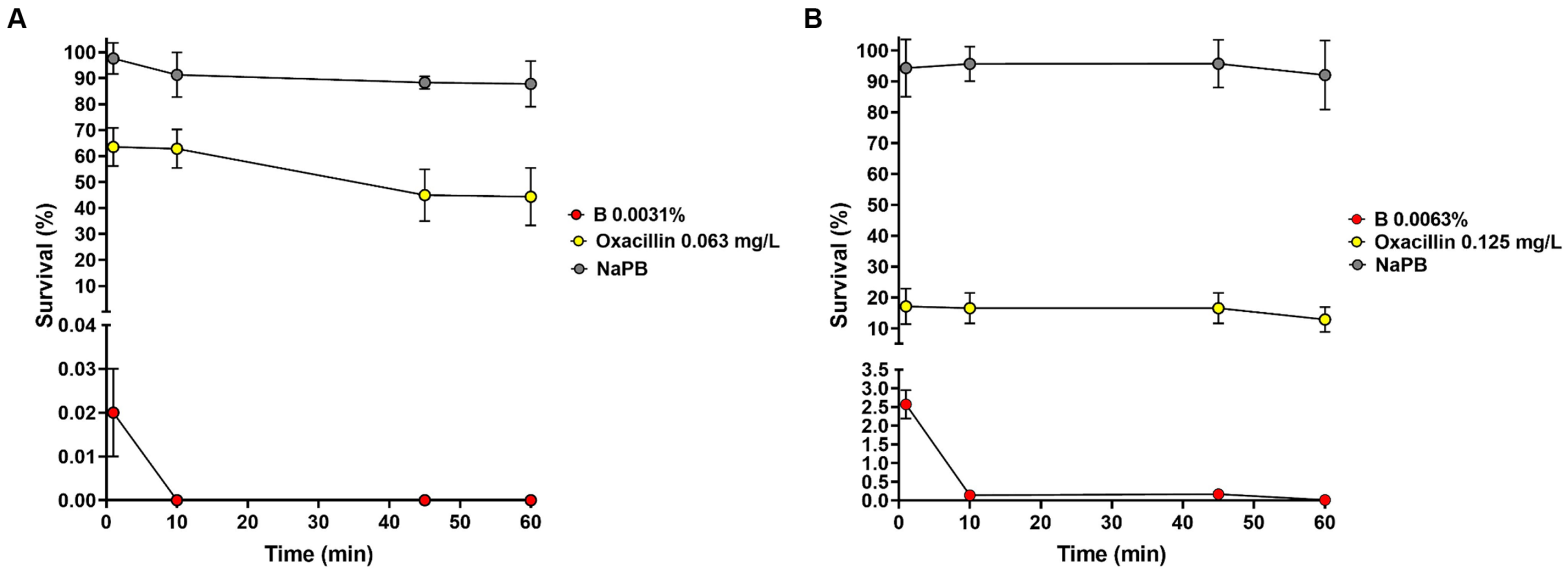
Effect of B at the MIC concentrations on the survival of *Staphylococcus aureus* ATCC 6538 **(A)** and *Staphylococcus epidermidis* CI-1 **(B)**. Min: minutes; NaPB (0.01 M Sodium Phosphate Buffer, pH 7.2): grey circles; B at the MICs: red circles; oxacillin at the MICs: yellow circles.

*Staphylococcus aureus* ATCC 6538 and *S. epidermidis* CI-1 were selected as model organisms for time-kill experiments and 1.05 ± 0.29 × 10^6^ CFU and 2.70 ± 0.89 × 10^6^ CFU, respectively, were inoculated in NaPB containing B at the MIC values. Bacteria inoculated in NaPB alone and in NaPB containing oxacillin at the MIC concentrations (i.e., 0.063 and 0.125 mg/L, respectively) were used as controls. After 1 min of incubation in the presence of B, *S. aureus* and *S. epidermidis* loads decreased to 1.79 ± 0.55 × 10^2^ CFU and to 6.75 ± 1.50 × 10^4^ CFU, respectively (Figures 1A,B). No residual living *S. aureus* cells were detected starting from 10 min of incubation, while the percentage of survival after 60 min of incubation for *S. epidermidis* CI-1 was 0.012 ± 0.009% (corresponding to 4.01 ± 0.84 × 10^2^ CFU). Taken together, these results indicate that B exerts rapid bactericidal effect against *Staphylococcus* species. When compared to oxacillin, B displayed more rapid bactericidal effects. In fact, after 60 min of incubation in the presence of oxacillin, *S. aureus* and *S. epidermidis* loads were found to be 4.44 ± 0.27 × 10^5^ and 3.23 ± 0.20 × 10^5^, respectively, corresponding to a survival rate of 44.36 ± 11.09% and 12.87 ± 4.08% (Figures 1A,B).

### 3.3 B did not show toxic effects on RBCs

To test cytotoxicity of the citrus extract, human RBCs were used as models (Farag and Alagawany, 2018). Percentages of 44.18 ± 9.11% and 9.18 ± 2.60% of hemolysis were obtained using B concentrations of 0.05 and 0.025%, respectively. At a concentration of 0.013% and lower, less than 1% hemolysis was observed. In particular, a 0.30 ± 0.17% and 0.23 ± 0.13% of hemolysis was registered using the MIC values of B against staphylococci (0.0063 and 0.0031%). No alterations in RBCs morphology and diameter due to B treatment were evidenced from Giemsa-stained slides. Overall, these findings indicate that active concentrations of the extract against *Staphylococcus* strains do not produce hemolysis on human erythrocytes.

### 3.4 The polyphenol mixture synergizes with ampicillin, methicillin, oxacillin, and meropenem against MRSA and MRSE strains

The interaction between B and antibiotics belonging to different classes (i.e., ampicillin, methicillin, oxacillin, meropenem, levofloxacin, gentamycin, chloramphenicol, daptomycin, and vancomycin) was tested by checkerboard assays. We chose to analyze this interaction also with meropenem since this drug is sporadically used for treating complicated skin infections due to staphylococci and methicillin resistance often correlates with resistance to carbapenems, to which meropenem belongs (Baldwin et al., 2008). The MIC values of B and antibiotics alone and in combination, as well as the calculated FICIs, are reported in Table 2. As indicated by the FICI values, synergistic activity of B with ampicillin, methicillin, meropenem, and oxacillin was evidenced only for strains resistant to these antibiotics (i.e., *S. aureus* ATCC 43300, *S. epidermidis* ATCC 35984, *S. epidermidis* CI-2). In fact, when the extract was tested in combination with methicillin, the MIC values of B were 16-, 4-, and 8-fold lower for *S. aureus* ATCC 43300, *S. epidermidis* ATCC 35984, and *S. epidermidis* CI-2, respectively. In addition, the MIC of methicillin was 4-fold reduced for *S. aureus* ATCC 43300 and *S. epidermidis* CI-2, and 8-fold reduced for *S. epidermidis* ATCC 35984. A reduction of 4- or 8-fold in the MIC values of ampicillin, meropenem, and B was observed for methicillin-resistant strains (i.e., *S. aureus* ATCC 43300, *S. epidermidis* ATCC 35984, *S. epidermidis* CI-2). A 4-fold reduction in the MIC of B and an 8- or 16-fold reduction in the MIC of oxacillin were evidenced against *S. aureus* ATCC 43300, *S. epidermidis* ATCC 35984, and *S. epidermidis* CI-2.

Interestingly, when experiments were performed using the antibiotic-susceptible *S. aureus* ATCC 6538 and *S. epidermidis* CI-1, no synergism was evidenced (FICI = 2). No interaction was evidenced between the extract and levofloxacin, gentamycin, chloramphenicol, daptomycin, and vancomycin for any of the tested strain (Table 2).

### 3.5 B causes membrane depolarization

**Figure 2:**
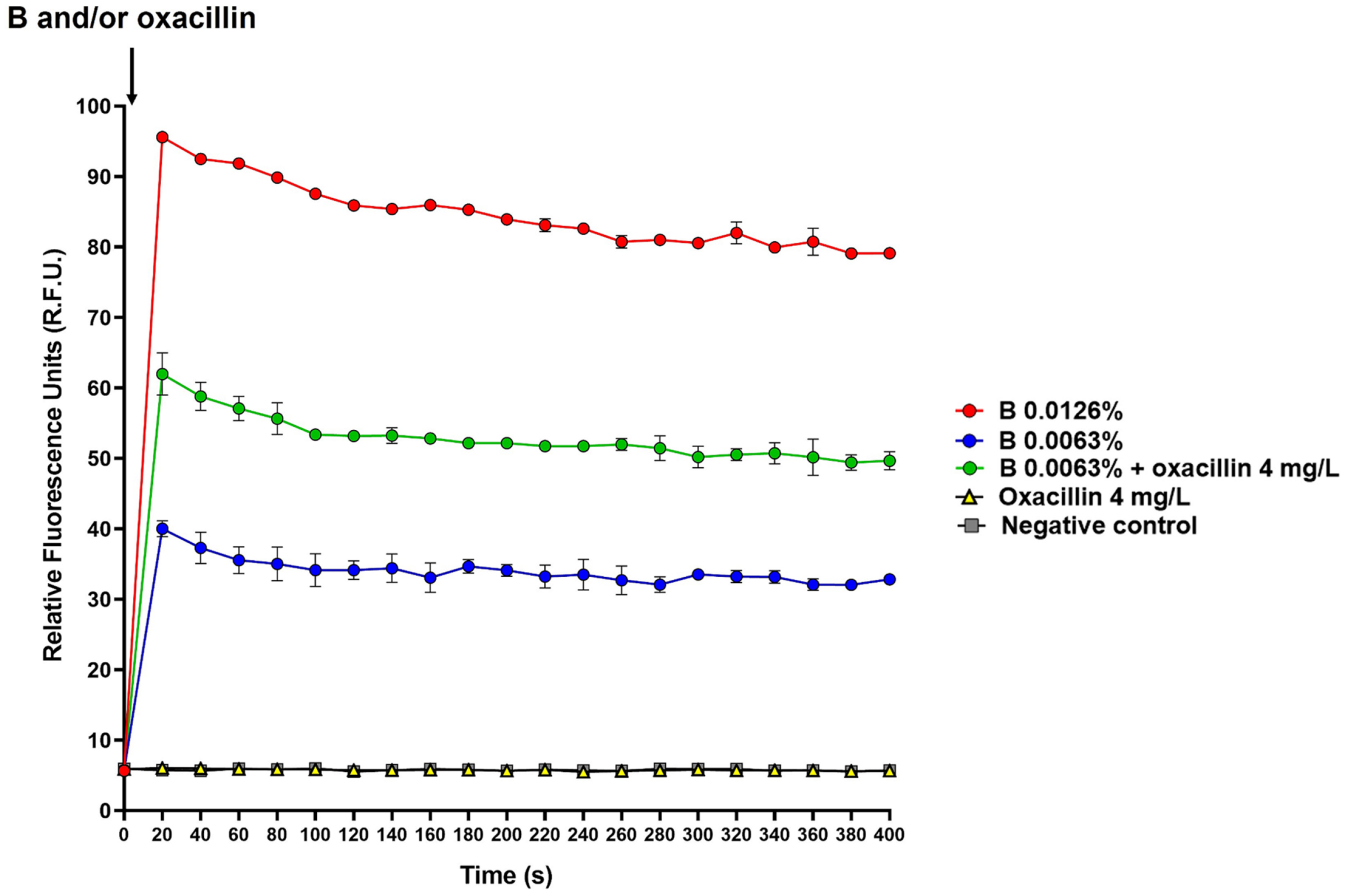
Effect of B alone and in combination with oxacillin on *S. aureus* ATCC 43300 membrane potential determined by the DiSC3(5) assay. The black arrow indicates B and/or oxacillin supplementation. DiSC3(5) fluorescence was expressed as relative fluorescence units (R.F.U.). Times up to 400 s were included in the figure. B at 2-fold the MIC (0.0126%): red circles; B at the MIC (0.0063%): blue circles; B at the MIC (0.0063%) in combination with oxacillin at the MIC (4 mg/L): green circles; oxacillin at the MIC (4 mg/L): yellow triangles; untreated cells (i.e., negative control): grey squares.

To investigate whether B, alone and in combination with oxacillin, could affect the membrane potential, the DiSC3(5) assay was used. Experiments were performed using *S. aureus* ATCC 43300, which was selected as model organism for methicillin-resistant strains. The intensity of fluorescence emitted by cells before DiSC3(5) supplementation was 0.026 ± 0.006 R.F.U., while immediately after the addition of DiSC3(5) peaked to 791.63 ± 17.45 R.F.U. Then, cells were incubated for 1 h at 37°C for enabling dye quenching, and fluorescence measured at this time point was used as time 0 in the assay (Figure 2). B alone (0.0063 and 0.0126%), oxacillin alone (4 mg/L), and 0.0063% B supplemented with 4 mg/L oxacillin were added to cell suspension and fluorescence started to be measured after about 20 s from antimicrobial supplementation and every 20 s for a total of 10 min. As shown in Figure 2, the dye fluorescence reached a peak within 20 s after the addition of B, thus indicating a very rapid effect of the polyphenol mixture in dissipating membrane potential. After this time, fluorescence was quite stable over time. In addition, DiSC3(5) fluorescence was found to be dependent on the B concentration. In fact, the highest values of fluorescence were obtained when B was used at 2-fold the MIC (i.e., 0.0126%).

Interestingly, when B was used in combination with oxacillin, DiSC3(5) fluorescence was found to be higher than that of B alone. In contrast, oxacillin alone had no effect on the membrane potential (Figure 2). This finding indicates that oxacillin reinforces the effect of B on the bacterial membrane.

### 3.6 Sequential exposure to sub-inhibitory B concentrations does not induce resistance in *Staphylococcus aureus*

To test whether the presence of sub-inhibitory concentrations of B could stimulate the emergence of resistance to the mixture, experiments of resistance development were performed using *S. aureus* ATCC 6538 as model organism for staphylococci. The MIC of B against *S. aureus* ATCC 6538 determined by the agar dilution assay was 0.0031%. The strain was propagated for 10 sequential transfers on plates containing 0.5-fold the MIC of B (0.0016%) to obtain a confluent growth at each transfer. At the 5th and 10th transfer, colonies were collected and seeded on plates containing 2-fold the MIC of B (0.0063%). No colonies with an increased MIC of B were obtained seeding about 10^10^ bacteria exposed to sub-inhibitory concentrations for 5 and 10 transfers, thus indicating that the proficiency of the extract to induce resistance is <10^−10^. In addition, bacteria collected at the 10^th^ transfer did not show cross-resistance to β-lactams. In fact, the MICs of ampicillin, methicillin, oxacillin, and meropenem were the same of those obtained for *S. aureus* ATCC 6538 (0.031, 0.5, 0.063, and 0.016 mg/L, respectively).

## 4 Discussion

Citrus is well known to be beneficial for human health and some polyphenols abundant in these fruits were isolated and individually tested for their antimicrobial effects (Adamczak et al., 2019; Koolaji et al., 2020; Manso et al., 2021; Miles and Calder, 2021; Pontifex et al., 2021; Naz et al., 2023). Nevertheless, the antibacterial activity of citrus extracts is poor studied. Some reports investigating the antimicrobial effect of different citrus peel and pulp extracts highlighted a broad-spectrum, even if variegated, antibacterial activity of these natural derivatives (Tomotake et al., 2006; Okeke et al., 2015; Fratianni et al., 2019; Ulhaq et al., 2021; Hasan et al., 2022; Li et al., 2022). Depending on the species and tissues subjected to extraction, citrus can profoundly differ in polyphenol composition and amount (Durand-Hulak et al., 2015; Wang et al., 2019). Therefore, it is presumable that different citrus extracts can exert different antimicrobial effects. This lack of homogeneity can lead to poor reproducibility and to difficulty in comparing the results obtained using different extracts. For this reason, in this study we chose to use B since possesses a controlled and homogeneous polyphenol composition even in different batches (U.S. Food and Drug Administration, 2013).

To the best of our knowledge, only three reports testing the *in vitro* activity of B against a limited number of bacteria, yeast, and molds are available (Cormier et al., 2013; Ben-Fadhel et al., 2019; Maherani et al., 2019). These studies focused on its use as surface cleaner or supported its application as food treatment in the food industry. The citrus extract is also included as active ingredient in a liposomal formulation commercialized as medical device for the ocular antisepsis (Vagge et al., 2021; Mencucci et al., 2022; Mazzantini et al., 2024). Nevertheless, evidence on the potential application of B in the clinical practice to treat staphylococcal infections is still lacking. Herein, we spotlight on the *in vitro* anti-staphylococcal activity of the citrus extract with the aim to test if it could be clinically useful for treating staphylococcal infections. To this regard, we believe that the use of mixtures of natural molecules with antibacterial effects may constitute an advantage in the clinical practice, since exerting a more powerful activity compared to single compounds and reducing the possibility to induce resistance.

We show that B exerts marked antibacterial effect against all the tested *Staphylococcus* strains, being effective at very low concentrations and displaying rapid bactericidal activity. This finding is concordant with the evidence that polyphenols, particularly flavonoids, are highly active against Gram-positive bacteria, due to the presence of hydroxyl groups that favor their interaction with the plasma membrane (Ben-Fadhel et al., 2019; Álvarez-Martínez et al., 2020; Nguyen and Bhattacharya, 2022; Oulahal and Degraeve, 2022). In addition, we believe that the anti-staphylococcal activity of B is strengthened by the presence of different polyphenols, which act altogether leading to rapid microbial cell death. Considering the powerful activity of the extract, cytotoxicity assessment was believed essential to exclude potential toxic effects on human cells. Although several biological models are available, erythrocytes represent simple, abundant, and cheap tools for cytotoxicity evaluation (Farag and Alagawany, 2018). Since polyphenols primarily act disrupting the plasma membrane, we chose to use the hemolysis assay to test B cytotoxicity (Pagano and Faggio, 2015; Podsiedlik et al., 2020). At concentrations ≤0.0125%, cytotoxicity of the extract was lower than 1%, supporting its safety when used at active dosages against *Staphylococcus* strains.

Some polyphenols were shown to synergize with traditional antibiotics, including β-lactams, fluoroquinolones, and macrolides against antibiotic-resistant staphylococci (Diarra et al., 2013; Ekambaram et al., 2016; Miklasińska-Majdanik et al., 2018; Alhadrami et al., 2020). Herein, we demonstrate a synergistic activity of B with the β-lactam drugs ampicillin, methicillin, meropenem, and oxacillin against methicillin-resistant strains. β-lactams exert their antimicrobial activity specifically targeting penicillin-binding proteins (PBPs) that catalyze cell-wall transpeptidation, thus inhibiting cell wall biosynthesis (Peacock and Paterson, 2015). In *S. aureus*, methicillin-resistance is predominantly due to the presence of the *mecA* gene encoding an alternative PBP (i.e., PBP2A) with reduced affinity for methicillin. The presence of *mecA* induces resistance toward benzylpenicillin, phenoxymethylpenicillin, ampicillin, amoxicillin, piperacillin, ticarcillin, methicillin, oxacillin, cephalosporins, and carbapenems (Vestergaard et al., 2019). This mechanism of resistance is due to the dislocation of the active-site serine in a narrow-extended cleft of the protein and to the presence of an allosteric site that regulates the exposure of the active site controlling substrate access (Peacock and Paterson, 2015; Alhadrami et al., 2020; Hou et al., 2023). Although further studies will be required to explain the synergistic activity of B with ampicillin, methicillin, oxacillin, and meropenem against MRSA and MRSE strains, we speculate that it is linked to the mechanism of methicillin-resistance in staphylococci, with two possible ways. First, some flavonoids like rutin, quercetin, and hesperidin have been predicted to efficiently interact with PBP2A, with rutin displaying the highest affinity for the enzyme (Rani et al., 2014; Alhadrami et al., 2020; Di Lodovico et al., 2020; Aribisala and Sabiu, 2022; Saini et al., 2024). Since these molecules are all contained in B, we hypothesize that they can interact with the active or allosteric site of PBP2A through hydrophobic or hydrogen bonds, thus causing conformational changes in the enzyme and increasing its accessibility to β-lactams. However, since the complete polyphenol composition of B is partially unknown, it is also possible that other polyphenols contained in the extract could downregulate the expression of *mecA*, affecting PBP2A synthesis (Chang et al., 2019; Lin et al., 2020).

Many flavonoids, like quercetin, rutin, and hesperidin, have been shown to act on the plasma membrane in bacteria (Iranshahi et al., 2015; Bouarab Chibane et al., 2019; Ivanov et al., 2022; Nguyen and Bhattacharya, 2022; Tagrida et al., 2023). In fact, the hydroxyl groups of polyphenols can directly interact with the bacterial plasma membrane by hydrogen bonding causing pores formation and membrane disruption. This interaction can also cause electron delocalization, thus affecting membrane potential (Bouarab Chibane et al., 2019). Membrane potential is determined by the diffusion of ions throughout ion channels and active pumps localized in plasma membrane. This diffusion is driven by the electrochemical potential difference of ions between the cytoplasm and the extracellular environment (Benarroch and Asally, 2020). Herein, the ability of B to affect membrane potential was verified by the DiSC3(5) assay. In fact, DiSC3(5) is a cationic potential-sensitive and self-quenching fluorescent dye that is incorporated in polarized membranes. When membrane is depolarized, DiSC3(5) is released and emits fluorescence (Benfield and Henriques, 2020). We show that B is able to depolarize the bacterial plasma membrane. In particular, the mixture dissipates membrane potential within 20 s from its supplementation, thus indicating a marked and very rapid depolarizing effect. In addition, oxacillin appears to increase the depolarizing activity of B. This can be due to the fact that the antibiotic, acting on the bacterial cell wall, favors the activity of the citrus extract on the membrane.

One of the most critical issues linked with the use of antimicrobials is the development of bacterial resistance, a phenomenon that compromises their use in the treatment of infections. For this reason, we investigated on the proficiency of B to induce the emergence of resistant strains in *S. aureus*, which was selected as model organism for staphylococci. Interestingly, no colonies resistant to a B concentration 2-fold higher than the original MIC value were found after ten transfers in the presence of sub-inhibitory concentrations of the extract. Although bacteria can develop resistance to polyphenols (Tabasco et al., 2011), the finding that B did not induced resistance in *S. aureus* can be linked to the nature of the extract that contains a complex mixture of polyphenols. The extract complexity could represent an obstacle for the emergence of resistance to all polyphenol molecules, thus supporting its potential use as anti-staphylococcal agent. In addition, prolonged exposure to B does not increase cross-resistance to β-lactams.

## 5 Conclusion

**Figure 3:**
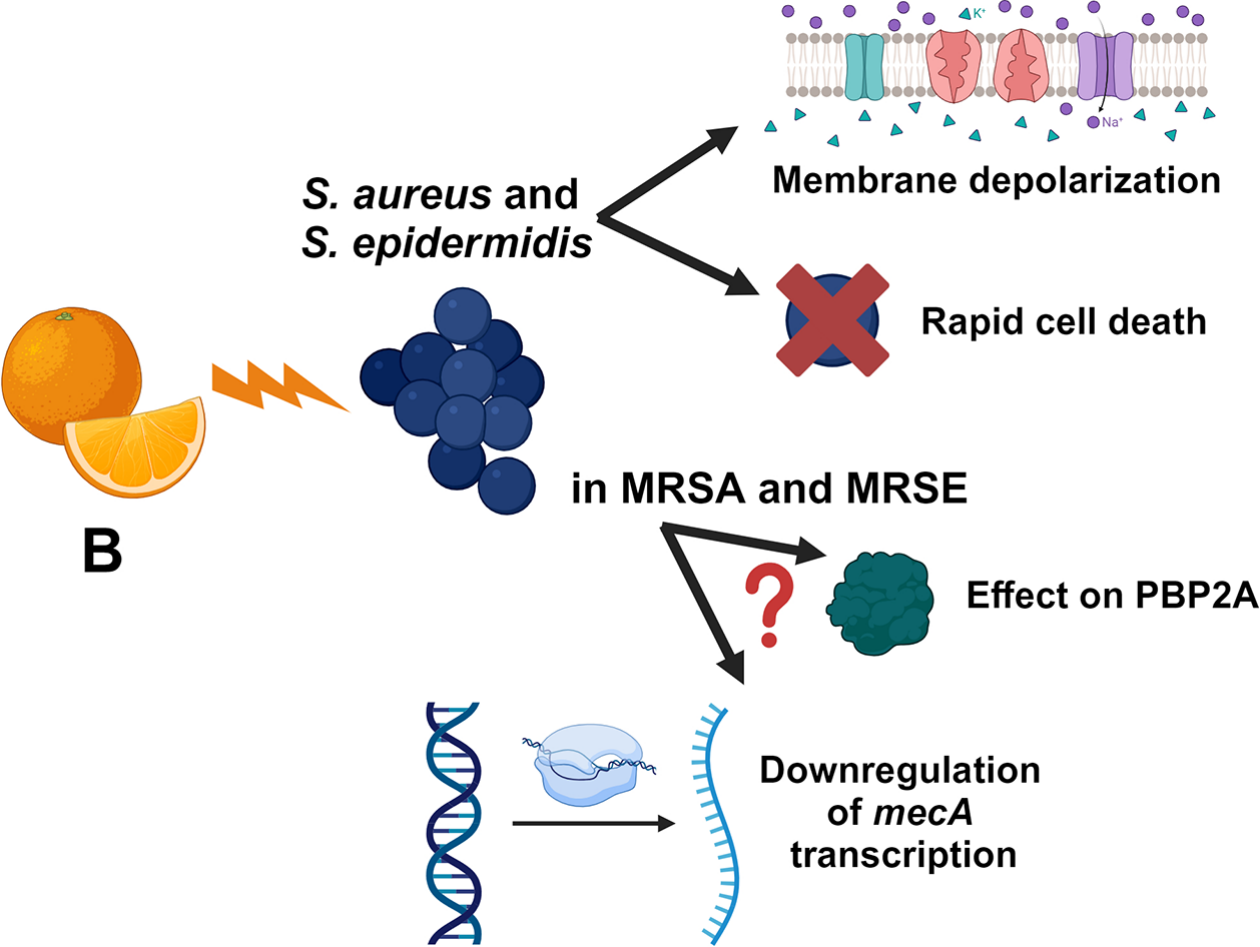
Schematic representation of the putative mechanisms of actions of B. The figure is created with BioRender.com.

Nowadays, antibiotic resistance represents one of the most serious threats for public health. Herein, we focused on the antimicrobial activity of the citrus-extract B against *Staphylococcus* species, demonstrating that the mixture exerts a powerful and rapid bactericidal effect on these microbes with no cytotoxic effects on human RCBs at active concentrations. We additionally show that B synergizes with methicillin, oxacillin, and meropenem against MRSA and MRSE strains and that the prolonged exposure to sub-inhibitory concentrations of the extract does not induce resistance in *S. aureus*. We demonstrate that B induces rapid depolarization of the bacterial plasma membrane in a concentration-dependent manner and that this depolarizing activity is powered in the presence of oxacillin. The putative mechanisms of action of B against staphylococci are represented in Figure 3. Although *in vivo* studies will be required to validate the activity of B during infections, we believe that this *in vitro* contribution encourages the use of the extract, alone or in combination with β-lactams, as potential anti-staphylococcal treatment.

## Data availability statement

The raw data supporting the conclusions of this article will be made available by the authors, without undue reservation.

## Author contributions

DM: Writing – review & editing, Writing – original draft, Validation, Methodology, Investigation, Formal analysis, Conceptualization. MM: Writing – review & editing, Investigation. MC: Writing – review & editing, Investigation. VR: Writing – review & editing, Investigation. FC: Writing – review & editing, Validation, Methodology, Investigation. AL: Writing – review & editing, Validation, Supervision. GB: Writing – review & editing, Validation, Supervision, Funding acquisition. EG: Writing – review & editing, Validation, Supervision, Methodology, Funding acquisition, Conceptualization.
